# Graphene Oxide/Polyethylene Glycol-Stick for Thin Film Microextraction of β-Blockers from Human Oral Fluid by Liquid Chromatography-Tandem Mass Spectrometry

**DOI:** 10.3390/molecules24203664

**Published:** 2019-10-11

**Authors:** Hanieh Karimiyan, Mohammad Reza Hadjmohammadi, Karthik Laxman Kunjali, Mohammad Mahdi Moein, Joydeep Dutta, Mohamed Abdel-Rehim

**Affiliations:** 1 Functional Materials Group, Department of Applied Physics, School of Engineering Sciences, KTH Royal Institute of Technology, Isafjordsgatan 22, Kista, SE-164 40 Stockholm, Sweden; karimiyanhanieh@gmail.com (H.K.); Laxman@kth.se (K.L.K.); joydeep@kth.se (J.D.); 2 Department of Chemistry, University of Mazandaran, Babolsar 47416-95447, Iran; hadjmr@umz.ac.ir; 3 Department of Radiopharmacy, Karolinska University Hospital, S-171 76 Stockholm, Sweden; mohammad.moein@sll.se; 4 Department of Clinical Neuroscience, Centre for Psychiatry Research, Karolinska Institutet, SE-171 76 Stockholm, Sweden

**Keywords:** graphene oxide/polyethylene glycol, wooden stick, β-blocker, oral fluid, LC-MS/MS

## Abstract

A wooden stick coated with a novel graphene-based nanocomposite (Graphene oxide/polyethylene glycol (GO/PEG)) is introduced and investigated for its efficacy in solid phase microextraction techniques. The GO/PEG-stick was prepared and subsequently applied for the extraction of β-blockers, acebutolol, and metoprolol in human oral fluid samples, which were subsequently detected by liquid chromatography tandem mass spectrometry (LC-MS/MS). Experimental parameters affecting the extraction protocol including sample pH, extraction time, desorption time, appropriate desorption solvent, and salt addition were optimized. Method validation for the detection from oral fluid samples was performed following FDA (Food and Drug Administration) guidelines on bioanalytical method validation. Calibration curves ranging from 5.0 to 2000 nmol L^−1^ for acebutolol and 25.0 to 2000 nmol L^−1^ for metoprolol were used. The values for the coefficient of determination (R^2^) were found to be 0.998 and 0.996 (*n* = 3) for acebutolol and metoprolol, respectively. The recovery of analytes during extraction was 80.0% for acebutolol and 62.0% for metoprolol, respectively. The limit of detections (LODs) were 1.25, 8.00 nmol L^−1^ for acebutolol and metoprolol and the lower limit of quantifications (LLOQ) were 5.00 nmol L^−1^ for acebutolol and 25.0 nmol L^−1^ for metoprolol. Validation experiments conducted with quality control (QC) samples demonstrated method accuracy between 80.0% to 97.0% for acebutolol and from 95.0% to 109.0% for metoprolol. The inter-day precision for QC samples ranged from 3.6% to 12.9% for acebutolol and 9.5% to 11.3% for metoprolol. Additionally, the GO/PEG-stick was demonstrated to be reusable, with the same stick observed to be viable for more than 10 extractions from oral fluid samples.

## 1. Introduction

According to the studies by the World Health organization (WHO), nearly 17.9 million people died from cardiovascular diseases in 2016, representing 31% of all global deaths [1]. Beta-blockers, acebutolol and metoprolol are an important clinical nitrogen containing-drugs which are regularly prescribed for treatment of various cardiovascular diseases such as hypertension, irregular heart rhythms, strokes, and angina pectoris [2]. In addition, β-blockers are known to improve a heart’s function by relaxing muscles and reducing the heart rate, an effect which is often misused by athletes competing in professional sports [3]. The International Olympic Committee and International Sports Federations thus have forbidden their usage in athletic competitions [3].

The measurement of drugs in biological samples has crucial importance for pharmaceutical companies in respect of drug discovery and development of new drugs. Various analytical techniques have been used for the analysis of β-blockers in biological samples [4,5,6,7,8,9,10,11]. Owing to the complexity of biological samples, sample preparation is of significant importance prior to analysis. Liquid-liquid extraction (LLE), solid phase extraction (SPE), and protein precipitations (PPT) are the common sample pretreatment techniques used in bioanalysis. However, these techniques are time consuming and labor intensive. Also, there are other serious drawbacks, such as high matrix background in sample extracts in PPT and the use of a large volume of polar solvents to form emulsion in LLE techniques [12]. Solid phase extraction has become the technique of choice for complex biological samples since the primary requirements of the extraction (matrix exchange, desalting, removal of the macromolecule, and high polar compounds) are compatible with the properties of the sorbent [12]. According to the aims of green chemistry, miniaturized sample preparation methods have been developed and introduced during the last two decades. Solid phase microextraction (SPME) is one of the popular and solvent-free sample preparation techniques introduced in 1990s [13]. Solid phase microextraction is widely used for chemical analysis in complex matrices due to its advantages such as simplicity, low cost, and versatility [14,15,16]. Thin film microextraction as a new mode of SPME was introduced in 2003 to improve its disadvantages, such as low extraction capacity [17]

In recent years, the development of nanomaterials, and their inherent large surface to volume ratios, has led to their application in bioanalysis. Graphene, a nanostructured two-dimensional monolayer structure of carbon atoms with sp^2^ hybridization and an ultra-high surface area, has shown considerable potential for SPE, compared to other carbon-based nanomaterials like carbon nanotubes [18,19]. Graphene oxide (GO), the oxidized form of graphene, consists of various oxygen functional groups on the basal and edge of the layer (mainly carbonyl, epoxy, and hydroxyl), which can lead to a variety of interactions like hydrogen bonding with organic materials [20]. Polyethylene glycol (PEG) is a well-known biocompatible polymer with numerous properties including high solubility in water and organic solvents, nontoxicity, high crystallinity, flexibility, and self-lubrication [21,22]. According to FDA approval, PEG is applicable in pharmaceutical companies as solvents, liquid delivery, and tablet fillers [23]. Since, there are different oxygen functional groups on the surface of graphene oxide, it can stabilize PEG through strong hydrogen bonds with ether groups in the molecular chain of the polymer, which leads to an effective and high surface PEG capacity and hence the ability to perform a wide range of interactions.

In this study we report the use of GO/PEG nanocomposite as an efficient adsorbent used in thin film micro extraction for the determination of acebutolol and metoprolol from human oral fluid. The proposed method is simple, convenient, fast, and affordable for the analysis of drugs in biological samples.

## 2. Experimental

### 2.1. Material and Instrumentation

Acebutolol hydrochloride, metoprolol tartrate, and pentycaine, used as an internal standards (I.S) were purchased from (Sigma-Aldrigh, St. Louis, MO, USA) (Figure S1). Graphene oxide (GO) was obtained from Voxco Pigment Pvt. Ltd. (Mumbai, India). Polyethylene glycol (PEG-20000), Acetonitrile, methanol, and formic acid (FA) were purchased from Sigma-Aldrich (Steinheim, Germany). The length and the thickness of the wooden sticks were 100 × 2 mm. Deionized water used for the mobile phase was produced by Milli-Q system (Millipore Co, Bedford, MA. USA). Samples were analyzed using a triple-quadrupole mass spectrometer, Z-spray Quattro-micro (Waters, Manchester, UK) with an electrospray ionization source (ESI). The system was equipped with a binary pump of LC10Advp from Shimadzu Corporation (Kyoto, Japan) and a CTC-PAL Analytics AG (Zwingen, Switzerland) autosampler with 25 µL sample loop. Chromatographic separation was carried out using a µBondapak C18, 10 µm. 3.9 × 150 mm dimension (Waters, Stockholm, Sweden). Acquisition and processing were performed using MassLynx (version 4.1, Waters, Manchester, UK).

### 2.2. Synthesis of GO/PEG Hybrid Material

**Figure 1:**
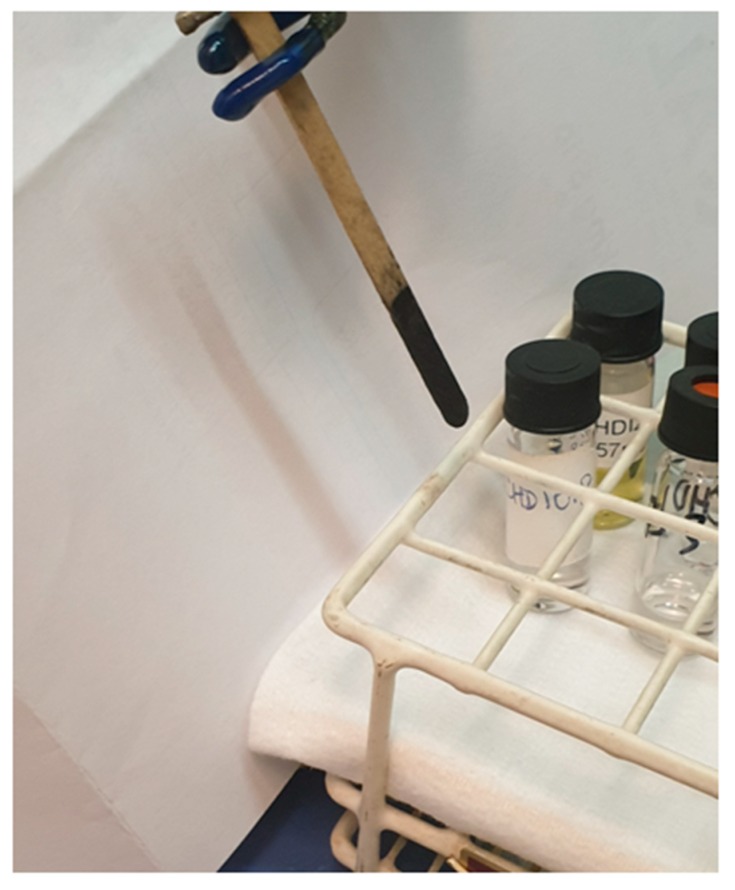
Coated graphene oxide/polyethylene glycol (GO/PEG)-Stick.

25.0 mg of GO powder was dispersed in 10.0 mL of deionized water in a 25.0 mL beaker and kept in an ultrasonic bath for 30.0 min at room temperature. Subsequently, 50.0 mg PEG was added to the above solution and the mixture solution was ultrasonicated for another 30 min. Fifteen wooden sticks were inserted in the GO/PEG solution and sonicated for 60 min. After sonication, the wooden sticks were removed from the beaker and carefully washed with distilled water to remove unattached PEG molecules. Approximately 1–2 cm of the sticks was covered by GO/PEG hybrid material. As a final step, the sticks were freeze dried overnight to stabilize the composite layer on their surface. The surface morphology and coating distribution of graphene oxide (GO) on the wooden stick was characterized using a ZEISS ULTRA55 field emission scanning electron microscope (FESEM) working at 3 kV. The samples were coated with a thin layer of gold nanoparticles to improve image resolution. Figure 1 shows the image of a wooden stick covered by GO/PEG hybride material which was utilized as the extraction device.

### 2.3. LC-MS/MS Conditions

The chromatographic conditioning was performed in gradient mode. Mobile phase A comprising of water/acetonitrile/formic acid (99.5:0.5:0.1) and mobile phase B comprising of acetonitrile/water/formic acid (80:20:0.1) were used. The gradient elution started with 10% of mobile phase B, which was increased to 90% within 5 min. The elution was maintained at 90% of mobile phase B for 4 min, following which, and after a delay of 6 min, elution was restored to 10% of mobile phase B. The mobile phase flow rate was 0.6 mL min^−1^. The MS source and desolvation temperature was set at 150 °C and 400 °C, respectively. Nitrogen (N_2_) was used as the nebulization gas and argon was chosen as the collision gas. The eluate from the column was directed into the electrospray ionization (ESI) source. Multiple reactions monitoring (MRM) was used to study the compounds with the following transitions for quantifications: 337.15→116.1 (acebutolol), 268.16→116.34 (metoprolol), and 303.3→154.3 (pentycaine). In Table 1 we have summarized the MS parameters used for the measurements of the analytes that were studied.

### 2.4. Stock Solutions and Sample Preparation Process

Two stock solutions of each analyte having concentrations of 200 µmol L^−1^ each were prepared by dissolving them in methanol and stored at 4 °C (one for standard solution and the other one for quality control (QC) solution). The standard solutions of acebutolol with concentrations of (5, 10, 50, 200, 600, 1000, 1500, and 2000 nmol L^−1^) and three QC samples (QCL (low): 15, QCM (medium): 900, and QCH (high): 1600 nmol L^−1^) were prepared from stock solutions in oral fluid. Moreover, the standards for metoprolol with the concentrations of (25, 50, 100, 250, 500, 1000, 1500, and 2000 nmol L^−1^) and QC samples (QCL: 75, QCM: 900, QCH: 1600 nmol L^−1^) were also prepared from the oral fluid samples. The human oral fluid samples (from 6 healthy volunteers) were centrifuged at 6000 rpm for 10 min, following which the supernatant was diluted 4-fold by deionized water. All of the solutions were prepared in diluted oral fluid.

The wooden sticks with an approximate length of 10 cm (with at least 1 cm coated with GO/PEG) were used for extraction. The GO-PEG coated sticks were ultrasonically cleaned in 1.0 mol L^−1^ hydrochloric acid solution for about 30 min and then in deionized water for 30 min in order to remove any contaminants before coating. Finally, all the sticks were dried in air. For each experiment, 1.0 mL of sample was added to a 2.5 mL sample vial. At least 1 cm of a GO/PEG-stick was inserted into the sample vial, after which the stick and sample vial were placed on a shaker for 10 min. Subsequently the sticks were removed and introduced into 1.0 mL methanol with 0.1% formic acid (FA) which acted as the desorption solvent, following which the vials were put on a shaker for 5 min. The wooden stick was then removed, and the extracted sample was evaporated in the presence of N_2_ and the residue was resolved in 200 µL of 50% (v/v) deionized water in acetonitrile and then 50 µL of extraction solution was injected directly into LC-MS/MS.

## 3. Result and Discussions

The use of graphitic material for microextraction by packed sorbent (MEPS) for the analysis of acebutolol and metoprolol has been investigated in a previously reported work from our group [24]. Graphitic material possesses a unique property to retain polar compounds, which act as suitable candidates for sorbents in SPE. In the present study, a novel adsorbent GO/PEG nanocomposite material has been used for thin film microextraction and has been successfully demonstrated for the extraction of acebutolol and metoprolol from human oral fluid samples via detection by LC-MS/MS. Different parameters affecting the method such as sample pH, extraction time, desorption time, and solution and salt addition were evaluated and optimized.

### 3.1. Structure of GO/PEG-20000 Hybrid Material

**Figure 2:**
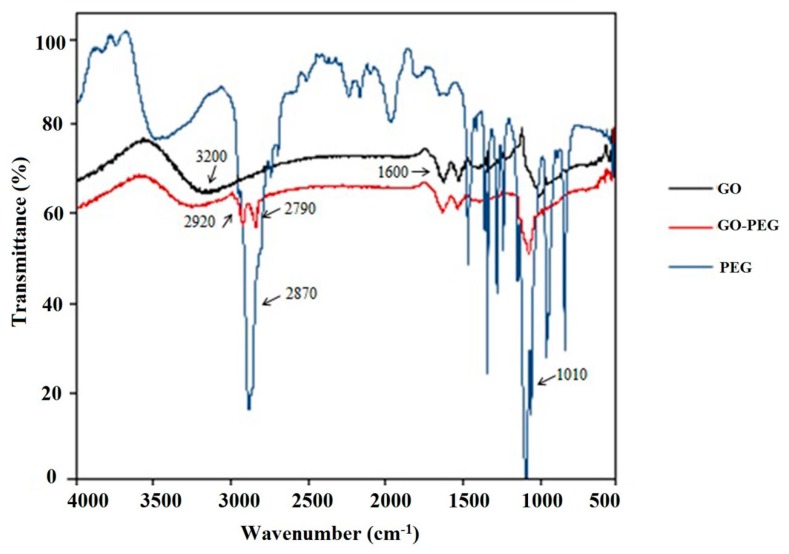
FT-IR spectrum of GO, GO/PEG-20000 and PEG-20000.

Figure S2 show the scanning electron micrograph (SEM) of a blank wooden stick (A,B), and a prepared stick covered with GO/PEG nanocomposite (C,D). As can be observed from the images, while the GO/PEG coating does not comprise of uniformly sized particles, there appears to be a well distributed and dense coating on the stick surface, which can make the surface effective for binding acebutolol and metoprolol. The dense coating is supported by, and further studied in, Figure 2, which shows the FT-IR spectrum of graphene oxide, PEG-20000, and GO/PEG. GO samples show a strong and broad peak around 3200 cm^−1^ corresponding to OH stretching as well as the carbonyl group (C=O) in COOH stretching absorption bands around 1600 cm^−1^. For the GO/PEG nanocomposites, the absorption peak at 3300 cm^−1^ related to OH stretching is observed, along with additional absorption peaks at 2920 cm^−1^ and 2790 cm^−1^ corresponding to CH_2_ stretching vibrations, and 1010 cm^−1^ absorption attributed to C-O-C vibration from the PEG molecules. The presence of these oxygen containing groups results from the formation of hydrogen bonds between graphene oxide and PEG ether groups.

### 3.2. Method Development

#### 3.2.1. Effect of Adsorption Time

**Figure 3:**
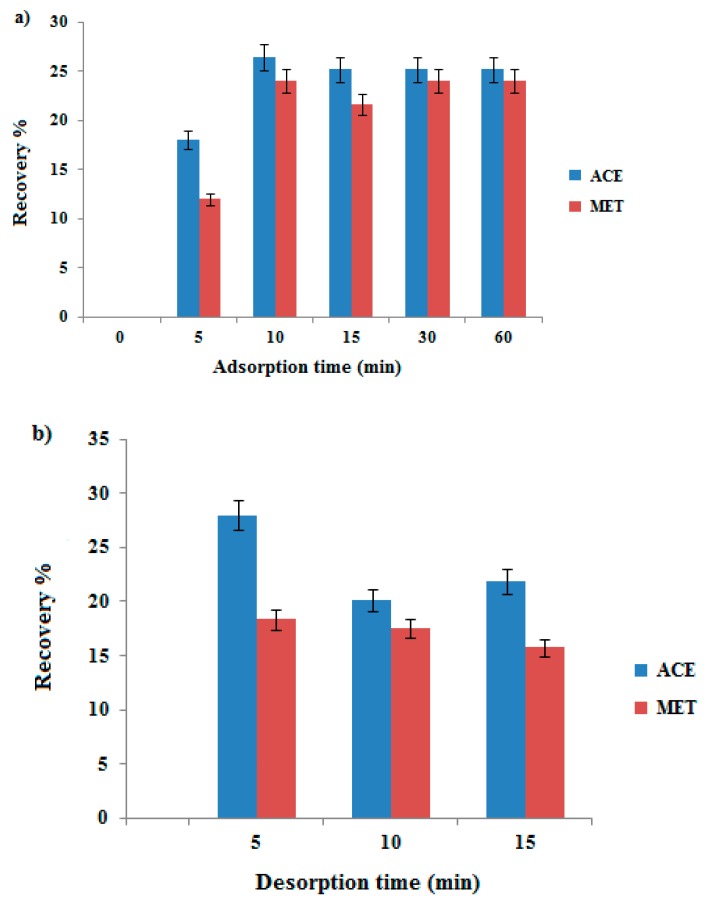
The effect of adsorption time (**a**) and desorption time (**b**) on the analyte response (given as extraction recovery) sample conc. 1000 nmol L^−1^.

In order to study the effect of adsorption time on extraction efficiency, adsorption periods ranging between 5 and 60 min were evaluated. Extraction recovery was observed to increase with adsorption time up to 10 min, and then remained stable thereafter as shown in Figure 3a. Based on these results, the adsorption process between the GO/PEG-stick and the analytes was found to depend on the contact time until the equilibrium was reached. Hence, 10 min of adsorption time was selected as the optimized time for extraction of analytes. In addition, ACE has two amide groups while MET has only one, thus the interaction of ACE to the sorbent should be better than MET resulting in a higher recovery of ACE compared to MET.

#### 3.2.2. Effect of Desorption Time

The effect of desorption time was evaluated in the range of 5–15 min by shaking and is shown in Figure 3b. The recovery % of analytes was found to increase with increasing desorption time up to 5 min. After this time the recovery % became slightly decreased until 15 min, which may be due to the distribution of the analytes in the sorbent, which can be reversible. As a result, desorption time of 5 min by shaking was chosen for further analysis.

#### 3.2.3. Effect of Sample Solution pH

The extraction recovery was studied at different pH values including physiological pH, acidic pH (by adding 0.1% FA), and basic pH (by adding 0.1% ammonium hydroxide). Acebutolol and metoprolol are weak bases, with pKa of 9.57 and 9.67, respectively [25]. The highest extraction recovery was obtained at pH = 7.0. Based on their pK_a_ values, these analytes exist as positive ions around pH 3.0–9.0 due to the protonation of the amino group, and turn into neutral molecules between pH 10.0–12.0 [26]. Wu et al. [27] has reported that the zeta potential of GO/PEG is −18.9 ± 1.9 mV, confirming that the surface of GO/PEG carries negative charges. Consequently, the electrostatic attractions besides the hydrogen bonds and the π–π interactions between the bulk π system of GO and analyte molecules containing the aromatic ring and double bond are the main interactions between GO/PEG sorbent and the analytes.

#### 3.2.4. Effect of Desorption Solution

Different desorption solutions were studied to achieve the highest desorption recoveries for the analytes. Methanol, acetonitrile, 0.1% FA in methanol, and 0.1% ammonium hydroxide in methanol were investigated. Results showed that using methanol with 0.1% FA as desorption solution achieved the highest desorption recovery. Methanol, with its polar and protic characteristics, was found to give the highest desorption recovery compared to acetonitrile, which is an aprotic solvent. Moreover, formic acid in methanol can have an effect on the functionality of the GO/PEG and also can interact with the analyte and facilitate its desorption, thus improving the recovery.

#### 3.2.5. Effect of Sample Ionic Strength

The effect of the salt addition to the sample solution was evaluated by adding sodium chloride in the range of 0.0–10% (*w*/*v*). The extraction recoveries of acebutolol and metoprolol decreased with increasing salt concentrations, which can probably be attributed to a viscosity increase of the sample solution and also the interactions between the analytes and salt ions that can occur. As a result, the movement of the analyte towards the sorbent got restricted with increasing salt concentration. Hence, all subsequent experiments were performed without the addition of salt.

#### 3.2.6. GO/PEG-Stick Extraction Capacity

**Figure 4:**
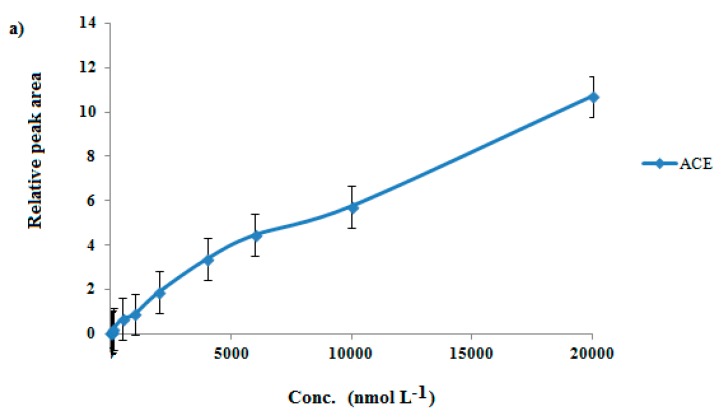

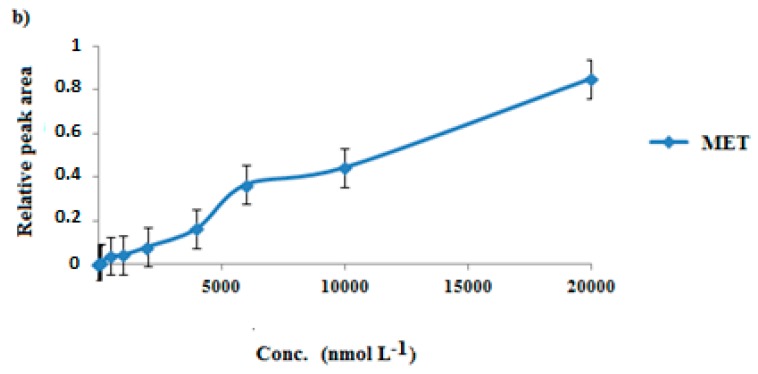
The capacity range of GO/PEG-stick for acebutolol (**a**) and metoprolol (**b**).

The capacity of the GO/PEG hybrid material was investigated in the range of 2–20,000 nmol L^−1^. Based on the results in Figure 4, the extraction was linear up to 6000 nmol L^−1^ for acebutolol and 4000 nmol L^−1^ for metoprolol. In higher concentrations, some of the adsorption sites of the GO/PEG nanocomposite layer became saturated and the extraction recovery was not linear.

#### 3.2.7. Selectivity of GO/PEG-Stick

**Figure 5:**
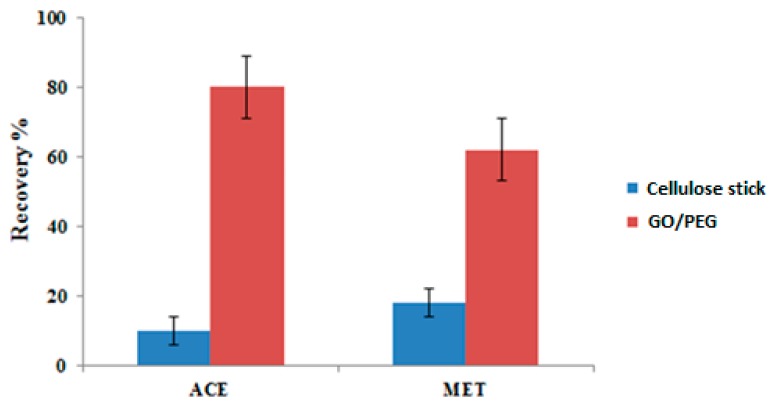
Efficiency of GO/PEG-stick compared to blank cellulose stick (1000 nmol L^−1^).

The extraction efficiency of the cellulose stick was investigated without any sorbent and compared with the efficiency after the stick was covered by GO/PEG nanocomposite adsorbent. In Figure 5 we show the extraction recoveries for the studied analyte, wherein it can be observed that the extraction recovery increased when the cellulose sticks were covered with GO/PEG nanocomposite sorbent. For acebutolol and metoprolol, the extraction recovery using GO/PEG-stick was approximately 4-fold higher compared to the blank wooden stick.

#### 3.2.8. Reusability of the GO/PEG-Stick

In order to investigate the reusability of the GO/PEG-stick, the used stick was washed with methanol after each extraction-desorption cycle and reused for the extraction of analytes. The same GO/PEG-stick could be used more than 10 times without any significant loss in extraction efficiency.

### 3.3. Method Validation

#### 3.3.1. Calibration, Accuracy, and Precision

Validation of the method was performed according to FDA guidelines [28]. Each calibration curve had eight standards (S1–S8) in the range of 5–2000 nmol L^−1^ for acebutolol and 25–2000 nmol L^−1^ for metoprolol. The QC samples at three different concentrations (high: H, medium: M and low: L) were prepared. Quadratic regression with a weighting factor of 1/X was used for calibration curves. Each assay contained standards and quality control samples at three concentration levels. The accuracy and precision for the QC samples were determined using three assays. The accuracy was calculated as the degree of deviation of the measured value from the true value and was found to be in the range of 80.0% to 97.0% for acebutolol and 95.0% to 109.0% for metoprolol. The precision or relative standard deviation (RSD) is expressed as the standard deviation of the measurements over their mean value in percentage. The interday precisions of QC samples in oral fluid were in the range of 2.0% to 12.3% for acebutolol and 4.7% to 10.7% for metoprolol. The results of accuracy and precision are summarized in Table 2.

#### 3.3.2. Limit of Detection and Quantification

**Figure 6:**
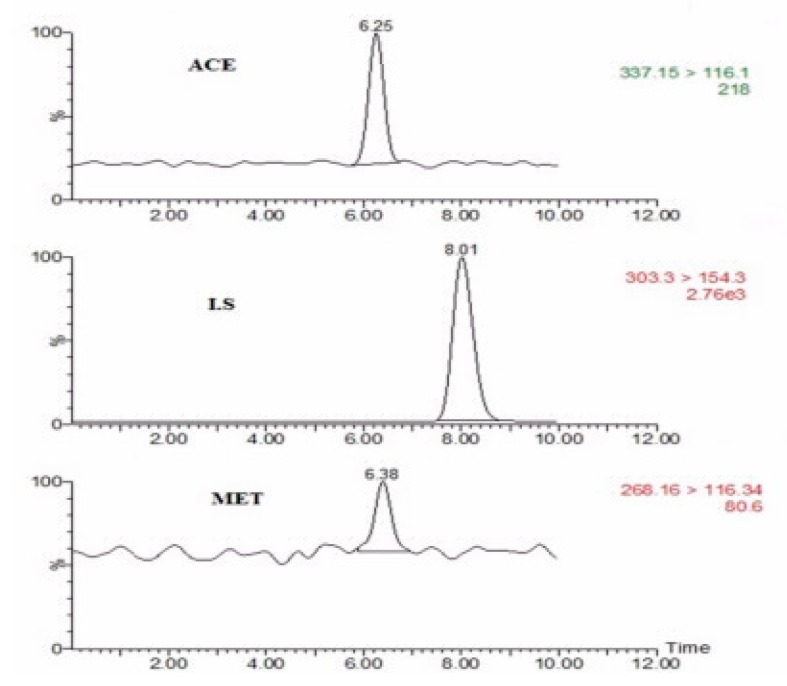
The chromatogram of lower limit of quantification (LLOQ) with internal standard.

The lower limit of quantification (LLOQ) and the limit of detection (LOD) are expressed as 10 × Sb/m and 3 × Sb/m, respectively (where Sb is the standard deviation of the blank and m is the slope of a calibration curve). The LOD for acebutolol and metoprolol were 1.25 and 8.00 nmol L^−1^, respectively. Also, the LLOQs were found to be 5.0 and 25.0 nmol L^−1^ for acebutolol and metoprolol. Figure 6 shows the LC-MS/MS chromatograms of the LLOQ for the analytes

#### 3.3.3. Carry-Over

The carry-over can arise from two major sources: the LC-MS system (injector and MS interface) and the extraction device. To decrease the memory effect of the extraction method, the sticks were washed with methanol for 15 min to minimize the carry-over. The carry-over was studied by injecting the extracted high standard solution (2000 nmol L^−1^) followed by the injection of the extracted blank sample. Typical carry-over was found to be 10% and 1.0% (blank peak area/LLOQ peak area) for acebutolol and metoprolol, respectively.

#### 3.3.4. Matrix Effect

The matrix effect (ME) of the obtained signal from the detector depends on analyte chemical structure, matrix environment, and sample concentrations, which could potentially and quite significantly influence the accuracy and precision of detection [29]. In order to evaluate the matrix effect, blank oral fluid samples were extracted at optimized conditions following the above method and the analytes were added to the extract at two different concentrations of QC samples (QCL, QCH). In this case, ME was calculated by comparing the signal of these solutions obtained from LC-MS/MS analysis to that of pure methanol with the same concentration of acebutolol and metoprolol. Results show that human oral fluid extract did not have noticeable effect on the detector signal (˂10.0%).

### 3.4. Method Comparison

The analytical performance of the GO/PEG-stick was compared with SPE, SPME, and MEPS for the analysis of acebutolol and metoprolol. The results in Table 3 shows that this method is comparable in terms of recovery, accuracy, and precision to the published methods. Also, this proposed method has a significantly higher extraction recovery compared to SPME (about 20 times).

## 4. Conclusions

To our knowledge, this is the first report on the application of graphene oxide/polyethylene glycol as a thin layer adsorbent on a cellulose stick for the microextraction and sensitive detection of two β-blockers in human oral fluid samples. The proposed method demonstrates high extraction recovery showing acceptable accuracy and precision for the studied analytes. This technique is fast, convenient, cost-effective, and requires small amounts of samples and solvents, similar to microextraction by packed sorbents (MEPS). Results obtained in this work show that the GO/PEG-stick can be used as a suitable sampling method for the analysis of trace levels of β-blockers in oral fluid samples of patients with cardiovascular diseases.

## Figures and Tables

**Table 1:** Optimized liquid chromatography tandem mass spectrometry (LC/MS/MS) parameters for the studied analytes.

Compound	Retention (mins ± SD)	Precursor Ion (*m*/*z*)	Product Ion (*m*/*z*)	Cone Energy (V)	Collision Energy (eV)
**Acebutolol**	6.25 ± 0.015	337.15	116.1, 141.14,	25	22
**Metoprolol**	6.38 ± 0.017	268.16	116.34, 177,33	30	20
**Pentycaine (I.S)**	8.01 ± 0.012	303.37	150.17, 154.3	35	20

**Table 2:** Accuracy and precision of the quality control (QC) samples in human saliva.

Compounds	Samples	Accuracy (%) (*n* = 12)	Precision (RSD%)	
			Intra-Day (*n* = 6)	Intra-Day (*n* = 12)
**Acebutolol**	**QCL**	97.0	12.3	12.9
	**QCM**	85.0	3.15	3.6
	**QCH**	78.0	2.0	8.7
**Metoprolol**	**QCL**	95.0	10.5	11.3
	**QCM**	101.0	4.7	9.5
	**QCH**	109.0	10.7	10.7

**Table 3:** Comparison of the proposed method with other extraction methods for the analysis of acebutolol and metoprolol. Microextraction by packed sorbents, MEPS; solid phase extraction, SPE; solid phase microextraction, SPME.

Method	Ex. Sorbent	Sample Volume		Ex. Yield (%)	Accuracy (%)	Matrix		Ref.
		mL	ACE	MET	ACE	MET		
LC-MS/MS	MEPS Polystyrene	0.1	50	50	98–104	94–100	Plasma	[30]
					103–107	91–99	Urine	
LC-MS/MS	SPE/ion exchange	2.0	84–96	-	84–96	-	Plasma	[31]
			>100		109–124	-	Urine	
LC-MS	In-tube SPME	1.0	5.0	2.4	82–89	72–76	Serum	[32]
					88–94	84–85	Urine	
LC-MS/MS	Stick/GO/PEG	1.0	80–90	62	80–97	95–109	Oral fluid	Present work

## Supplementary Materials

The following are available online, Figure S1: Chemical structures of β-blockers and internal standard, Figure S2: SEM image of cellulose stick (A,B) and GO/PEG-stick (C,D).
