# Supplementary material for: Graphene Oxide/Polyethylene Glycol-Stick for Thin Film Microextraction of β-Blockers from Human Oral Fluid by Liquid Chromatography-Tandem Mass Spectrometry
Source: Molecules. 2019 Oct 11;24(20):3664. doi: 10.3390/molecules24203664 (PMC6832871; doi:10.3390/molecules24203664)
Supplement: Supplementary file 1 [file molecules-24-03664-s001.pdf]

Supplementary Material

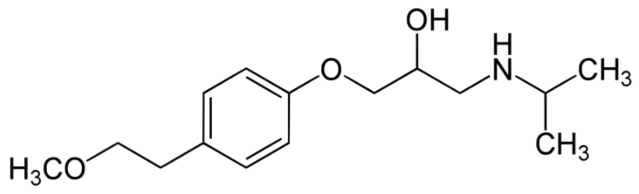

Metoprolol

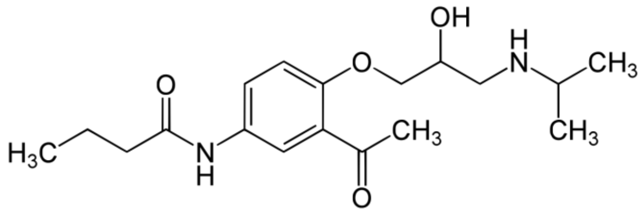

Acebutolol

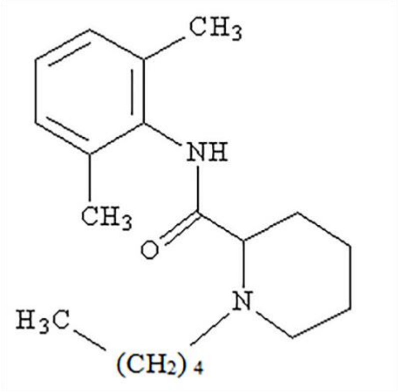

Pentycaine (I.S)

Figure S1. Chemical structures of β-blockers and internal standard

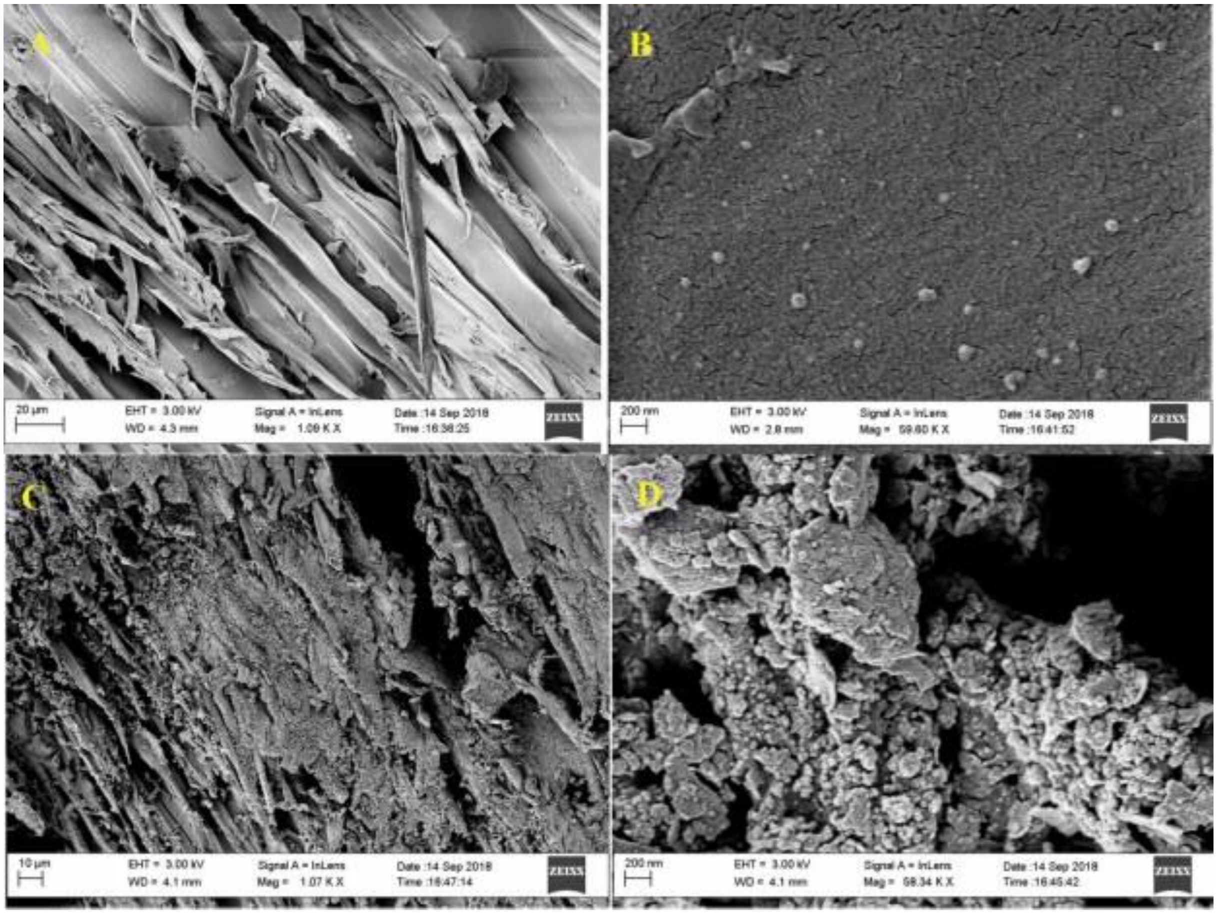

Figure S2. SEM image of cellulose stick (A,B) and GO/PEG-stick (C,D).
